# Reported Systems Changes and Sustainability Perceptions of Three State Departments of Health Implementing Multi-Faceted Evidence-Based Fall Prevention Efforts

**DOI:** 10.3389/fpubh.2017.00120

**Published:** 2017-06-08

**Authors:** Matthew Lee Smith, Ellen C. Schneider, Imani N. Byers, Tiffany E. Shubert, Ashley D. Wilson, Samuel D. Towne, Marcia G. Ory

**Affiliations:** ^1^Department of Health Promotion and Behavior, The University of Georgia College of Public Health, Athens, GA, United States; ^2^Department of Health Promotion and Community Health Sciences, Texas A&M School of Public Health, College Station, TX, United States; ^3^Center for Health Promotion and Disease Prevention, University of North Carolina at Chapel Hill, Chapel Hill, NC, United States; ^4^The University of Georgia School of Social Work, Athens, GA, United States; ^5^Shubert Consulting, Chapel Hill, NC, United States

**Keywords:** systems change, sustainability, fall prevention, older adults, evidence-based programs, intervention, evaluation

## Abstract

Although the concepts of systems change and sustainability are not new, little is known about the factors associated with systems change sustaining multi-state, multi-level fall prevention efforts. This exploratory study focuses on three State Departments of Health (DOH) that were awarded 5-year funding from the Centers for Disease Control and Prevention to simultaneously implement four separate yet related evidence-based fall prevention initiatives at the clinical, community, and policy level. The purpose of this study was to examine changes in partnerships and collaborative activities that occurred to accomplish project goals (examining changes in the context of “before funding” and “after funding was received”). Additionally, this study explored changes in State DOH perceptions about action related to sustainability indicators in the context of “during funding” and “after funding ends.” Findings from this study document the partnership and activity changes necessary to achieve defined fall prevention goals after funding is received, and that the importance of sustainability indicator documentation is seen as relevant during funding, but less so after the funding ends. Findings from this study have practice and research implications that can inform future funded efforts in terms of sector and stakeholder engagement necessary for initiating, implementing, and sustaining community- and clinical-based fall prevention interventions.

## Introduction

There is an ethical paradox that exists with providing extramural funding to introduce health promotion interventions in a community. When services are introduced into the community, health-related benefits are typically seen, but then the funding ends and the services are no longer available. The instability of funding may actually discourage communities from offering the services in the first place. Despite the promise of community benefit, often there is no opportunity for the initiative to continue unless local organizations can embed the intervention into their ongoing operations and offer the intervention as a routinized service.

Falls and fall-related injuries among older adults are a growing public health in the US. Falls among the aging population can lead to premature mortality, loss of physical functioning, loss of independence, and insurmountable financial burdens (1), with about one in every three adults over age 65 years falling each year (2). The impacts of injurious falls on individual health and well-being, interpersonal networks, and healthcare costs are widely recognized (3). As such, government agencies are initiating efforts to introduce multi-level, evidence-based fall prevention strategies in communities that engage a diverse array of health professionals, organizations, and stakeholders.

To avoid community disappointment and ill will toward funders, a recent trend in public health and aging services is to solicit grantees who can (1) evoke systems change by creating networks of health organizations and introduce policy to have lasting effects and (2) demonstrate the potential for sustainability through strategic long-term planning, innovative business acumen, and leveraged funding (4). Examples include recent funding solicitations from national agencies such as the Centers for Disease Control and Prevention (CDC) or the Administration for Community Living (ACL) for states to deploy systems thinking to tackle the challenge of fall prevention for older adults.

The goal of deploying systems thinking is to sustainably bring these programs to scale by focusing on relationship building between individuals and organizations across traditional disciplines (5). Systems changes are activities, procedures, and/or policy changes that occur within an organization; changes in relationships between organizations; or community-level changes that influence healthcare systems, legislation, regulations, or awareness efforts. In recognition of the value of systems change to fuel sustainable efforts, ACL included a specific goal of “Build[ing] partnerships … and identify[ing] innovative funding arrangements that can support these evidence-based falls prevention programs, while embedding the programs into an integrated, sustainable evidence-based prevention program network” in a recent funding announcement (4).

Although the concepts of systems change and sustainability are not new (6–8), little is known about the factors associated with systems change that occur over the course of a funding period to implement and hopefully sustain multi-state, multi-level fall prevention efforts. While funding is often provided to grantees to implement a single intervention, fewer grantees are charged with concurrently implementing multiple interventions in their service areas (9). Furthermore, while many studies examine the outcomes associated with individual interventions, there is less focus on process and changes necessary to accomplish grant objectives from the perspective of the grantees (10). This exploratory study focuses on three State Departments of Health (DOH) that were awarded 5-year funding from the CDC to simultaneously implement four separate yet related evidence-based fall prevention initiatives at the clinical, community, and policy level. The purposes of this exploratory study were to: (1) identify systems changes related to the types of fall prevention partners and stakeholders working with the State DOH from before receiving funding to after the funding was received; (2) examine systems changes related to the types of involvement in which sectors engaged with the State DOH from before receiving funding to after the funding was received; (3) identify policy and organizational changes that occurred as a result of receiving funding; and (4) assess the State DOH’s perceptions about and action related to sustainability indicators after the funding was received (thinking toward the future). This study contributes to the literature in that it identifies partnership and infrastructure changes that occurred to accomplish project goals (examining changes in the context of “before funding” and “after funding was received”). We believe this element can help communities when planning to introduce new interventions to their community. We hypothesize that State DOH will expand their partnerships, collaborations, and activities as a result of funding. We also hypothesize that State DOH perceptions and actions related to sustainability will change when thinking about efforts post-funding.

### State Falls Prevention Project (SFPP)

In 2011, the CDC initiated the SFPP, a 5-year project in which State DOH in Colorado (CO), New York (NY), and Oregon (OR) were funded to simultaneously implement four fall prevention strategies. Three of these strategies were evidence-based fall prevention programs [i.e., Tai Chi: Moving for Better Balance (TCMBB), Stepping On, and Otago Exercise Program (OEP)], each selected for their documented effectiveness to prevent falls in randomized control trials. Tai Chi and Stepping On are typically offered in community settings, and the OEP is delivered by physical therapists in a clinical setting. The fourth strategy was a clinical intervention to engage physicians and other clinicians in fall risk management through use of the CDC STopping Elderly Accidents Deaths and Injuries (STEADI) tool kit. Within each state, these four clinical and community fall prevention strategies were implemented in specific geographic areas based on greatest population need (e.g., population density, fall rates, and fall-related hospitalizations). Implementation sites and service areas were determined by each state grantee, as outlined in their grant applications. While the funded agencies were the State DOH, they were encouraged to create sustainable partnerships with other sectors (as they typically do) to engage participants, identify and train leaders, and deliver the interventions.

### Tai Chi: Moving for Better Balance

Tai Chi: Moving for Better Balance is a group-based exercise program intended to engage adults over age 65 years in eight Tai Chi forms to improve strength, balance, and physical performance (11–13). The program meets three times per week, 1 h each session, over 24 consecutive weeks. During the 24-week program, participants focus on weight shifting, postural alignment, coordinated movements, and synchronized breathing. These activities are low-impact and increase in difficulty as the workshop progresses.

### Stepping On

Stepping On is a group-based program that uses adult education and self-efficacy principles to engage community-dwelling older adults at risk of falling, those with a fear of falling, and/or those with a history of falls (13–16). The program is intended to increase self-confidence to make decisions and change behaviors in situations that may increase fall-related risk. The program meets 2 h per week, once a week, over seven consecutive weeks. A home visit (or follow-up telephone call) and booster session are also conducted.

### The OEP

The OEP is a one-on-one innovative model of low frequency physical therapy sessions delivered in the homes of frailer older adults (17–20). The program consists of a series of five leg-strengthening and 12 balance-retraining exercises that become progressively more difficult as the participant becomes stronger. The program is delivered by a physical therapist. Over an 8-week period, the participant receives four in-home sessions (i.e., an initial visit, a visit after 1 week, a visit 2 weeks thereafter, and a visit 4 weeks thereafter).

### The STEADI Tool Kit

The STEADI tool kit is a collection of materials, guidelines, and an algorithm intended to guide clinicians’ fall-related screening, treatment, and referral activities in clinical settings with their older adult patients (21). In support of promising clinical approaches to reduce falls (22) and interpreting practice guidelines of the American and British Geriatrics Societies (23), STEADI was developed by fall content experts and researchers at the CDC Injury Center (24). Contents of STEADI, as well as supplemental resources, can be found online (22).

To evaluate this effort, the Falls Evaluation Technical Assistance team was established and worked collaboratively with CDC Injury Prevention Staff and State Fall Prevention Program leads. The evaluation also included partnerships with large university-based academic institutions. As with most well-conceived interventions, a pilot phase (approximately 2 years) was incorporated so that materials and processes could be tested and modified prior to grand-scale, full implementation. More about the challenges, modifications, and lessons learned related to the pilot findings can be found elsewhere (25). Because the fall prevention programs were at different stages of development, the rollout was necessarily staggered (25–27). An evaluation plan was initially established that identified short- and long-term goals and objectives for this multi-state, multi-level intervention approach. Primary foci included developing the infrastructure for community and clinical programs and assessing the relative impact of each effort.

While the SFPP included multiple individual interventions, the overall aim of the effort was to support states to simultaneously implement these interventions in community and clinical settings to: (1) create a delivery infrastructure necessary for disseminating these programs in their respective geographic region(s); (2) form new partnerships to expand participant reach and program adoption; (3) evoke a systems change to collectively address falls across sectors and at multiple levels within their respective geographic region(s); and (4) consider opportunities to leverage funds and continue partnerships to ensure sustained program delivery post-funding.

## Materials and Methods

Data used for this study were collected using two cross-sectional internet-delivered surveys. Both surveys were distributed to the state leads (i.e., the primary point of contact and Principal Investigator for the grant) at the CO, NY, and OR State DOH. State leads were asked to complete the instruments within a 2-week period. In an effort to capture a comprehensive view from each State DOH, state leads were encouraged to invite project staff from their State DOH to provide input, which enabled each State DOH to collectively complete the questionnaires (i.e., only one survey instrument was submitted per State DOH). State DOH were encouraged to consult their administrative records when appropriate to ensure accurate and timely responses. Participation was voluntary, and participants were provided with an information sheet prior to completing the surveys. Institutional Review Board approval was received by Texas A&M University, the University of North Carolina—Chapel Hill, and the University of Georgia for all study activities. Details about the items included in these questionnaires are presented in Tables 1–5 in this study.

**Table 1:** **Sectors serving as partners for fall prevention activities**.

	Before funding			With funding		
	CO	NY	OR	CO	NY	OR
**Area agency on aging/senior center**						
State Unit on Aging	X	X	X	X	–	X
Area Agencies on Aging		X	X	√	X	X
Senior Centers			X	√	√	X

**Educational institution**						
Academic Institutions	X	X	X	X	–	X
Geriatric Education Centers			X			X
Area Health Education Centers			X			X

**Faith-based organization/philanthropic**						
Faith-based organization	X	X	X	–	X	X
Philanthropic foundation			X			X

**Healthcare organization**						
Physician offices			X	√	√	X
Emergency departments			X			X
Home health agencies	X	X	X	X	–	X
Hospitals			X	√		X
Integrated healthcare systems	X			X	√	√
Trauma centers			X	√		X
Veterans Administration Medical Centers	X	X	X	X	–	X
Rural Practice Network	X	X	X	–	–	X
Healthcare insurance agencies (e.g., Humana, Kaiser Permanente)	X	X	X	X	–	X

Local/county or other related health department organizations						
Local health department		X	X	√	X	X
County health department	–	X	X	√	X	X
Injury Community Planning Group	X	X	X	X	–	X
Injury data registries	X	X	X	X	–	X

**Multi-purpose/recreational organization/library**						
YMCAs	X	X	X	X	X	X
Parks and recreational organization	X	X	X	X	X	X
Library	X	X	X	–	–	X

**Residential care facility**	X	X	X	–	X	X
**Tribal center**			X			X
**Workplace**	X	X	X	–	–	X

*Blank = did not occur before or with funding; X = occurred before and with funding; √ = occurred with funding but not before funding; – = occurred before funding but not with funding*.

**Table 2:** **Stakeholders engaged in fall prevention activities**.

	Before funding			With funding		
	CO	NY	OR	CO	NY	OR
College or university faculty or staff	X	X	X	X	–	X
Community health workers		X			X	√
Older adults		X		√	X	
Physician champions			X	√	√	X
Physical therapists		X	X	√	X	X
Policy makers			X		√	X
Volunteers			X		√	X

*Blank = did not occur before or with funding; X = occurred before and with funding; √ = occurred with funding but not before funding; – = occurred before funding but not with funding*.

**Table 3:** **Types of sector involvement for fall prevention activites**.

	Before funding			With funding		
	CO	NY	OR	CO	NY	OR
**Exchange information with at least quarterly on fall prevention activities**						
Area Agency on Aging/Senior Center	X	X	X	X	X	X
Educational institution	X	X	X	X	–	X
Faith-based organization						√
Healthcare organization	X		X	X	√	X
Local/county health department	X		X	X	√	X
Multi-purpose/recreational organization/library				√	√	√
Residential care facility						√
Tribal center			X			X
Workplace						√

**Jointly plan activities with at least quarterly on fall prevention activities**						
Area Agency on Aging/Senior Center					√	√
Educational institution	X	X		–	–	√
Faith-based organization						√
Healthcare organization				√		√
Local/county health department				√	√	√
Multi-purpose/recreational organization/library				√	√	√
Residential care facility						√
Tribal center			X			X
Workplace						√

**Share resources with at least quarterly on fall prevention activities**						
Area Agency on Aging/Senior Center	X	X	X	X	X	–
Educational institution	X	X		X	–	√
Faith-based organization						√
Healthcare organization	X			X	√	√
Local/county health department	X	X		X	X	
Multi-purpose/recreational organization/library				√	√	√
Residential care facility						√
Tribal center			X			X
Workplace						√

*Blank = did not occur before or with funding; X = occurred before and with funding; √ = occurred with funding but not before funding; – = occurred before funding but not with funding*.

**Table 4:** **Policy and organizational systems changes (since the being funded)**.

	Since being funded		
	CO	NY	OR
Falls Prevention Awareness Day was adopted	X		X
Organizational plans have included falls prevention goals and activities	X	X	X
Organizations have signed Memorandums of Agreement concerning falls prevention activities			X
Legislators have taken actions to promote fall prevention			
Organizations have adopted models of training leaders and instructors in community fall prevention programs	X	X	X

**Table 5:** **Perceived importance of the usefulness of collecting sustainability indicators**.^a^

	During funding				After funding ends			
	Mean	CO	NY	OR	Mean	CO	NY	OR
# of organizations that have implemented new policies to sustain program delivery	8.00	10^b^	5	9	3.00	5	3	1
# of policies deployed at local level	7.33	10^b^	5	7	3.00	5	3	1
# of policies deployed at regional level	4.33	7^b^	5	1	3.00	5	3	1
# of policies deployed at state level	5.33	10^b^	5	1^b^	4.67	10	3	1
# of healthcare systems actively implementing fall prevention programs	9.00	10^b^	8	9^b^	3.67	7	3	1
# of healthcare systems implementing significant systems change to include clinical integration (systems that have integrated into EHR)	6.33	10^b^	8	1^b^	2.00	2	3	1
# of healthcare systems implementing significant systems change to include centralized referral systems	7.67	8^b^	8	7^b^	2.00	2	3	1
# of systems in place to efficiently connect older adults to classes	8.67	10^b^	8	8	2.67	4	3	1

*^a^Measured on a scale of 1 (not important) to 10 (extremely important)*.

*^b^Indicates that State DOH is currently collecting information about this sustainability indicator*.

The first survey was deployed approximately 2 years after funding was initially received. It assessed systems changes related to fall prevention in their respective states/service areas as a result of receiving this CDC funding. Participants were asked to complete a series of questions related to partners, stakeholders, activities, and policy/organization changes related to fall prevention activities. First, participants were asked to report the community sectors acting as partners for fall prevention activities [e.g., Area Agency on Aging (AAA)/senior centers, educational institutions, faith-based organizations/philanthropic, healthcare organizations, and residential facilities]. Most sectors were further delineated to gain specific information related to partnerships for fall prevention. Next, participants were asked to report the stakeholders engaged in fall prevention activities (e.g., college or university faculty or staff, older adults, physician champions, and physical therapists). Next, participants were asked to report the ways in which each sector worked with the State DOH for fall prevention. For each sector, participants were asked to report if they did the following activities related to fall prevention at least on a quarterly basis: (1) exchanged information; (2) jointly planned activities; and (3) shared resources. Participants were asked to report information twice for items related to partners, stakeholders, and activities. The first was about their current partners, stakeholders, and activities (after funding was received). The second was retrospectively about their partners, stakeholders, and activities before funding was received. Finally, participants were asked to report whether or not specific policy and organizational systems changes were initiated since being funded [e.g., Falls Prevention Awareness Day (FPAD) was adopted, organizational plans have included falls prevention goals and activities]. Following the survey, 1-h in-depth interviews were conducted with state grantee leads (colleagues and staff) to gain clarification and additional context related to their survey results. However, these qualitative data are not presented in the current study.

The second survey was deployed approximately 4 years after funding was initially received. It assessed perceptions about the importance of sustainability indicators and current tracking of such sustainability indicators. First, participants were asked to rate the usefulness of collecting eight sustainability indicators (e.g., number of organizations that implemented new policies to sustain program delivery, number of policies at the local, regional, and state level, number of healthcare systems actively implementing fall prevention programs). Responses were scored on a scale of 1 (not important) to 10 (extremely important). As with the first survey, participants were asked to rate these items two times. Once was from the perspective of the sustainability indicator’s usefulness while being funded (during funding). Another was from the perspective of the sustainability indicator’s usefulness after the funding ends. Next, participants were asked to indicate whether or not they were currently collecting each of the eight sustainability indicators (e.g., organizations implementing new policies, policies deployed at various levels, and systems change in healthcare systems).

## Results

Table 1 presents sector involvement as partners to State DOH for fall prevention activities before funding and after funding was received. As can be seen, there was state-based variation of initial partnerships before receiving funding with all states reporting involvement from each sector, but OR reported more overall partnerships. Universally, partnerships across states before funding included State Units on Aging, academic institutions, faith-based organizations/philanthropic, home health agencies, Veterans Administration medical centers, rural practice networks, healthcare insurance agencies, injury community planning groups, injury data registries, YMCAs, parks and recreational organizations, libraries, residential care facilities, and workplaces.

After funding was received, changes in sector partnerships for fall prevention were observed. Most notably were changes in partnerships in the AAA/Senior Center and Healthcare organization sectors. More specifically, CO and NY formed partnerships with senior centers and physician offices. CO formed partnerships with AAAs, hospitals, and trauma centers. NY formed partnerships with integrated healthcare systems. CO also reported new partnerships with local and county health departments. As some partnerships were gained, others were discontinued after receiving funding. Most notably were changes in NY with partnerships for fall prevention discontinued with AAAs, academic institutions, home health agencies, VA medical centers, rural practice networks, healthcare insurance agencies, injury community planning groups, injury data registries, libraries, and workplaces. In CO, partnerships for fall prevention were discontinued with faith-based organizations/philanthropic, rural practice networks, libraries, residential care facilities, and workplaces.

Table 2 presents stakeholder engagement for fall prevention activities before funding and after funding was received. Before funding was received, all states reported college or university faculty/staff as stakeholders engaged in fall prevention activities. NY and OR reported more overall stakeholders engaged before funding was received. Many new stakeholders were engaged after funding was received. More specifically, in CO, older adults, physician champions, and physical therapists were engaged in fall prevention activities after funding was received. In NY, physician champions, policy makers, and volunteers were engaged in fall prevention activities after funding was received. In OR, community health workers were engaged in fall prevention activities after funding was received.

Table 3 presents types of sector involvement in State DOH fall prevention activities before funding and after funding was received. When asked about exchanging information with sectors on a quarterly basis, all three states reported this type of involvement with AAA/Senior Centers and educational institutions before funding was received. Furthermore, CO and OR reported this type of involvement with healthcare organizations and local/county health departments before funding was received. New quarterly information exchanges were reported after funding was received. After funding was received, CO reported exchanging information quarterly with multi-purpose/recreational organizations/libraries. After funding was received, NY reported exchanging information quarterly with healthcare organizations, local/county health departments, and multi-purpose/recreational organizations/libraries. NY reported no longer exchanging information quarterly with educational institutions after funding was received. After funding was received, OR reported exchanging information quarterly with faith-based organizations/philanthropic, multi-purpose/recreational organizations/libraries, residential care facilities, and workplaces.

When asked about jointly planning activities with sectors on a quarterly basis, few sectors were identified at baseline. CO and NY reported jointly planning activities quarterly with educational institutions before funding was received. OR reported jointly planning activities quarterly with educational institutions before funding was received. New quarterly jointly planned activities were reported after funding was received. After funding was received, CO reported jointly planning activities quarterly with healthcare organizations, local/county health departments, and multi-purpose/recreational organizations/libraries. After funding was received, NY reported jointly planning activities quarterly with AAA/Senior Centers, local/county health departments, and multi-purpose/recreational organizations/libraries. After funding was received, OR reported jointly planning activities quarterly with all sectors. After funding was received, CO and NY no longer reported jointly planning activities quarterly with educational institutions.

When asked about sharing resources with sectors on a quarterly basis, all three states reported this type of involvement with AAA/Senior Centers before funding was received. CO and NY reported sharing resources quarterly with educational institutions and local/county health departments before funding was received. CO reported sharing resources quarterly with healthcare organizations before funding was received. OR reported sharing resources quarterly with tribal centers before funding was received. New quarterly resource sharing was reported after funding was received. After funding was received, CO reported sharing resources quarterly with multi-purpose/recreational organizations/libraries. After funding was received, NY reported sharing resources quarterly with healthcare organizations and multi-purpose/recreational organizations/libraries. After funding was received, OR reported sharing resources quarterly with all sectors, with exception of AAA/Senior Centers. After funding was received, NY no longer reported sharing resources quarterly with educational institutions. After funding was received, OR no longer reported sharing resources quarterly with AAA/Senior Centers.

Table 4 presents policy and organizational systems changes reported by State DOH after funding was received. After funding was received, all three states reported that organizational plans included falls prevention goals and activities and that organizations adopted models of training leaders and instructors in community fall prevention programs. CO and OR reported that FPAD was adopted after funding was received (NY was already observing FPAD before funding was received). After funding was received, OR reported that organizations signed Memorandums of Agreement concerning falls prevention activities.

Table 5 reports State DOH perceptions of importance about the usefulness of collecting sustainability indicators before and after funding was received. On average, perceived importance scores ranged from 4.33 to 9.00, with dramatic variability across states. On average, before funding was received, the sustainability indicators that were viewed as most important included documenting the number of healthcare systems actively implementing fall prevention programs (*M* = 9.00), the number of systems in place to efficiently connect older adults to classes (*M* = 8.67), and the number of organizations that have implemented new policies to sustain program delivery (*M* = 8.00). On average, before funding was received, the sustainability indicators that were viewed as least important included documenting the number of policies deployed at the regional (*M* = 4.33) and state levels (*M* = 5.33). On average, after funding was received, importance scores for these sustainability indicators decreased, ranging from 2.00 to 4.67. While variability across states was observed for sustainability importance scores after funding was received, the variation was less than what was observed before funding was received.

On average, after funding was received, the sustainability indicators that were viewed as most important included documenting the number of policies deployed at the state level (*M* = 4.67) and the number of healthcare systems actively implementing fall prevention programs (*M* = 3.67). On average, after funding was received, the sustainability indicators that were viewed as least important included documenting the number of healthcare systems implementing significant systems changes to include clinical integration (*M* = 2.00) and centralized referral systems (*M* = 2.00).

Table 5 also reports the sustainability indicators collected by State DOH after funding was received (currently at the time of this study). Several differences were observed. CO reported currently collecting all sustainability indicators, NY reported currently collecting no sustainability indicators, and OR reported collecting half (4 of 8) of the sustainability indicators.

## Discussion

This study examined systems changes and sustainability indicators related to the rollout of a multi-state, multi-level fall prevention initiative. Results confirm hypotheses that as a result of funding, State DOH expanded their partnerships, collaborations, and activities as well as changed their perceptions related to post-funding sustainability. Findings from this exploratory study have practice and research implications that can inform future funded efforts in terms of sector and stakeholder engagement necessary for initiating, implementing, and sustaining community- and clinical-based fall prevention interventions. This study presents a unique case that included programs, states, academic institutions, and other key stakeholders. However, similar partnership structures between academic institutions, State DOH, and federal funders (i.e., the CDC) have also demonstrated success, thereby furthering the strong case for engaging multiple stakeholders (28). Thus, the current study may serve as a model to other similar multi-state, multi-component funding arrangements, while also highlighting that tailored strategies will be needed depending on settings, stakeholders, interventions, target population, and other factors.

Findings from this study document the partnership and activity changes necessary to achieve defined fall prevention goals after funding is received and that the importance of sustainability indicator documentation is seen as relevant during funding, but less so after the funding ends. This information can be of critical importance, given that funders value the sustained benefit of dollars invested in community health promotion efforts (29). Understanding ways to increase the perceived value of tracking or demonstrating sustained processes (e.g., communication between agencies) may be of use in demonstrating the long-term value of relatively short-term investments and activities as part of deliverables to funders. Identifying mechanisms for measuring sustained benefits is a challenge given engaging grantees post funding is complex, especially when other new or existing priorities are present. Thus, the focus on developing sustainability plans at the time of funding and continuing to update these plans and incorporate them into final reporting requirements is a reasonable option. However, long-term follow-up about the realized outcomes of such planning is an option that funders may consider by providing additional incentives for evaluation. Furthermore, identifying tools and models for evaluating the maintenance or sustainability of programs is essential. For example, TCMBB has been evaluated using the RE-AIM framework (30). As seen using this robust framework, an emphasis on Maintenance may reinforce sustainability of program implementation. In the current study, the intent for long-term maintenance was captured as a site intent to continue to provide TCMBB after the program ended.

Findings also highlight the importance of funding agencies emphasizing the need for dedicated evaluation expertise to accompany any large, multi-component initiative involving multiple sites (in this case states) (31). Integrating evaluators in grand-scale dissemination efforts have benefits including and transcending the provision of technical assistance during the funding cycle. Having the ability to capture and disseminate key measures of success is crucial for federal partners and other key stakeholders (e.g., community partners, academic partners, and policy makers). The utility of having evaluators involved early in the process allows for adjustment and tailoring of evaluation tools to help ensure that appropriate (e.g., valid and reliable) instruments are used. In addition, the ability to engage key stakeholders throughout the entire process encourages discussion of key metrics that are most valuable to all stakeholders. Furthermore, being aware of and incorporating (where appropriate) metrics that are of interest to policy makers (e.g., cost savings) can better guide the strategic dissemination of findings and recommended practices/procedures once evaluation activities are completed.

Although this study focused on fall prevention activities deployed through State DOH, these major findings transcend fall prevention and have applicability to other health issues (e.g., chronic disease, substance abuse, and sexual and reproductive health) and sectors (e.g., aging, healthcare, and faith-based). Activities that include partnership building, communication, reporting, and evaluation are not specific to fall prevention. Thus, lessons learned in terms of transferable activities can be used as a model for other similar projects.

It is well known that multi-level, multi-factorial efforts are most effective to evoke change at the individual level that distally impact community health (32); however, such efforts often require changes in existing infrastructure and practices. In the current study, states maintained many initial partnerships across sectors and were able to develop new partnerships after funding was received to better align efforts with the sectors/organizations that typically serve older adults and those at risk for falling. For example, based on the SFPP goals, partnerships created after funding was received were most notable in AAA/senior centers and healthcare organizations. The ability to form these new partnerships may not have been possible without the funding. Furthermore, partnerships that were discontinued after funding was received highlight the importance of focused efforts to maximize efficiency in terms of intervention-related training, embedment, and participant recruitment. For example, based on the SFPP aims, partnerships discontinued after funding was received were most notable in rural practice networks, libraries, and workplaces. The decisions to discontinue these partnerships may have been based on factors including the geographic service areas within states (more urban in nature) or the ability to reach older adults in these settings. In this context, it should also be acknowledged that grantees were State DOH, which may have influenced the types of partners and stakeholders engaged over time based on existing relationships and associated policies. It should also be noted that responses were self-reported, often retrospectively or hypothesized based on future events; thus the accuracy of these accounts may be biased.

Findings from this study suggest the importance of early and ongoing sustainability planning to guide partnership development, cultivation, and maintenance processes. While it is assumed that sustainability and partner selection are considered during the grant proposal development stage, pending the specific criteria associated with the request for proposals/applications designated by the funding agency, it is recommended that these aspects should be emphasized to grantees as requisite elements for intervention success. Partnerships should be purposively and critically selected based on the goals of the project and the unique strengths and attributes the partners/stakeholders can offer (including their ability to reach, recruit, and retain intervention participants) (33). Furthermore, the role each partner will play in the initiative should be well-conceived and discussed with transparency before the intervention begins (i.e., receives funding). As was seen in the current study, types of involvement across sectors increased on a quarterly basis after funding was received (i.e., exchanging information, jointly planning activities, and sharing resources). Changes observed in these types of interactions suggest greater partnership depth and quality, which can be leveraged for sustainability after the funding ends. As such, it is recommended that an environmental scan of existing local partners and organizations should be performed to identify suitable partners (with missions aligned with the grants’ purpose) and the potential of their inclusion to foster sustained efforts after the funding ends. For example, as reported by a State DOH grantee post-survey completion, a strategic partnership with a major insurance company created a referral system that enabled connections between physicians, older adults, and community-based fall prevention programs. Such a referral system, partially rooted in financial incentives, has potential to impact systems change and increase the likelihood of sustained fall prevention efforts in the local intervention delivery area (34). Although these types of referral systems are largely untested in terms of sustained efforts, this partnership strategy is encouraged and should be further examined in future multi-level initiatives.

In initiatives including the simultaneous introduction and delivery of multi-faceted fall prevention efforts, each intervention should not be assumed to roll out and diffuse at the same rate. For example, because many of the states funded in the SFPP already had experience implementing community-based fall prevention interventions (e.g., A Matter of Balance), the creation of adequate delivery infrastructures to offer Stepping On and TCMBB may have occurred more rapidly based on their understanding of training requirements/expectations and existing partnerships. Conversely, Otago and STEADI were newly introduced to the US (and therefore the grantees) during the SFPP (19, 35). Thus, the natural evolution of associated training, implementation, and evaluation requirements in the first few years of funding may have hindered rapid delivery and diffusion. Furthermore, the need for State DOH to engage and partner with new organizations in healthcare settings (e.g., physician offices and physical therapists) took more substantial time and effort. Now that many lessons have been learned and disseminated about the integrated multi-state rollout of these fall prevention strategies from the SFPP (13, 16, 25, 27), it is recommended that future funding include prescriptive suggestions and strategies for engaging new partners and their associated roles as well as ample resources and technical assistance pertaining to delivery infrastructure, implementation processes, and evaluation.

### Engaging Healthcare As a Model for Sustaining Fall Prevention Efforts

This study is unique in its examination of systems change among three states who were charged with simultaneously implementing four new fall prevention solutions in their communities. However, a variety of existing recommendations and resources exist to assist communities and grantees to prepare, execute, and sustain their evidence-based program dissemination efforts for older adults. For example, the National Council on Aging (NCOA) provides tool kits, webinars, and other resources about offering evidence-based program (see https://www.ncoa.org/center-for-healthy-aging/basics-of-evidence-based-programs).

Based on the timing of this initiative, the three State DOH included in this study should be considered pioneers of an evolving funding environment in the US. As a model to sustain these efforts, innovative financial agreements and partnerships must be established and expanded. Ideally, fall prevention efforts should be embedded in healthcare systems, hospitals, and trauma centers because they have access to older adults, trained professionals to screen for risk, facilities to provide services, and financial resources to support ongoing delivery. In the US, an important step toward sustaining fall prevention would be to have Medicare Advantage Plans and Providers pay for services (36, 37). Results and lessons learned from these pioneering states have influenced funding priorities nationally to build upon leveraging efforts with healthcare systems and new payment/funding/reimbursement structures (38).

As previously mentioned, the target population (i.e., older adults) and clinical interventions (i.e., Otago and STEADI) required State DOH to expand healthcare-related partnerships (and the type of sector involvement) to adequately serve participants and meet grant goals. While systems changes were observed, progress was not immediate and challenges were encountered (25, 39). However, State DOH are uniquely positioned to capitalize on their existing relationships with healthcare systems through epidemiological surveillance systems and task forces (40). While relationships are evolving and advancing between State DOH and healthcare systems, additional efforts are needed to nurture these partnerships for the purposes of embedding and sustaining fall prevention in healthcare settings. As such, the NCOA has established *learning collaboratives* to assist communities to work with healthcare for the purposes of supporting evidence-based programs for older adults (not just for fall prevention purposes) (41). Findings from this study highlight the importance of expanding partnerships by engaging more stakeholders in the systems change and sustainability processes. Such expansion can help facilitate dialog necessary to negotiate new funding arrangements with healthcare systems.

## Conclusion

While this study collected information related to State DOH’s perceptions about the importance of documenting sustainability indicators during times of funding and after funding ends, as well as sustainability indicators that were collected during the SFPP funding, data were not collected after funding concluded to identify ongoing action related to sustainability indicator tracking/monitoring. As seen in this study, the perceived importance of collecting sustainability indicators decreased after funding ends, which is not surprising (without funding, there is little incentive for grantees to continue evaluating their activities). To this end, it is recommended that future funding opportunities include extended evaluation efforts beyond grantee implementation funding to facilitate complete and comprehensive process and outcome evaluations and the lasting impacts of funded initiatives. It is recommended that future efforts work with grantees to identify and collect systems change and sustainability metrics specific to the intervention (e.g., program delivery and participant enrollment) and those that occur naturally and are publically available (e.g., new or modified policies).

Findings from this exploratory study show the influence of funding to bring about systems changes related to partnerships, stakeholders, and policies. Although these changes have potential to contribute to ongoing changes for fall prevention in these communities, the ability to document sustained efforts after funding ends is greatly diminished and largely unknown. To build upon the strengths and opportunities offered by funded fall prevention efforts, it is recommended that potential grantees begin formulating and rethinking new and existing partnerships for fall prevention to include rich and innovative interactions, collaboration, and fund leveraging. It is recommended that once funding is received, grantees become (or remain) involved in their State Fall Prevention Coalitions and consider forming new coalitions and task forces to band together local partners and guide local initiatives (42–44). Through formal collaborations comprised of diverse partners with a common focus, communities have a better chance of securing funding for fall prevention, meeting predetermined goals of funded multi-level interventions, serving older adults across sectors, and sustaining efforts after the funding ends.

## Ethics Statement

Institutional Review Board approval was received from Texas A&M University, the University of North Carolina—Chapel Hill, and the University of Georgia for all study activities. Details about the items included in these questionnaires are presented in Tables 1–5 in this study.

## Author Contributions

MS acquisitioned the data, conceptualized the study, performed data analyses, and drafted the manuscript. ES, TS, and MO acquisitioned the data and critically revised the manuscript. IB drafted the manuscript. AW and ST critically revised the manuscript.

## Conflict of Interest Statement

The authors declare that the research was conducted in the absence of any commercial or financial relationships that could be construed as a potential conflict of interest.
